# Automatic Segmentation of Epidermis and Hair Follicles in Optical Coherence Tomography Images of Normal Skin by Convolutional Neural Networks

**DOI:** 10.3389/fmed.2020.00220

**Published:** 2020-06-04

**Authors:** Rocío del Amor, Sandra Morales, Adrián Colomer, Mette Mogensen, Mikkel Jensen, Niels M. Israelsen, Ole Bang, Valery Naranjo

**Affiliations:** ^1^Instituto de Investigación e Innovación en Bioingeniería, I3B, Universitat Politècnica de València, Valencia, Spain; ^2^Department of Dermatology, Bispebjerg Hospital, University of Copenhagen, Copenhagen, Denmark; ^3^DTU Fotonik, Department of Photonics Engineering, Technical University of Denmark, Kongens Lyngby, Denmark

**Keywords:** skin OCT, follicular structures, layer segmentation, epidermis, convolutional neural networks, pilosebaceous unit

## Abstract

Optical coherence tomography (OCT) is a well-established bedside imaging modality that allows analysis of skin structures in a non-invasive way. Automated OCT analysis of skin layers is of great relevance to study dermatological diseases. In this paper, an approach to detect the epidermal layer along with the follicular structures in healthy human OCT images is presented. To the best of the authors' knowledge, the approach presented in this paper is the only epidermis detection algorithm that segments the pilosebaceous unit, which is of importance in the progression of several skin disorders such as folliculitis, acne, lupus erythematosus, and basal cell carcinoma. The proposed approach is composed of two main stages. The first stage is a Convolutional Neural Network based on U-Net architecture. The second stage is a robust post-processing composed by a Savitzky-Golay filter and Fourier Domain Filtering to fully define the borders belonging to the hair follicles. After validation, an average Dice of 0.83 ± 0.06 and a thickness error of 10.25 μ*m* is obtained on 270 human skin OCT images. Based on these results, the proposed method outperforms other state-of-the-art methods for epidermis segmentation. It demonstrates that the proposed image segmentation method successfully detects the epidermal region in a fully automatic way in addition to defining the follicular skin structures as main novelty.

## 1. Introduction

Optical coherence tomography (OCT) is a well-established imaging modality used to capture various aspects of biological tissues (1). OCT has been routinely used in ophthalmology due to the ease of light in penetrating ocular structures (2). In addition, as OCT is a non-invasive technique and therefore, does not require direct contact with the eye, it allows the *in-vivo* study of internal ocular structures such as the retina without performing any perforation.

**Figure 1:**
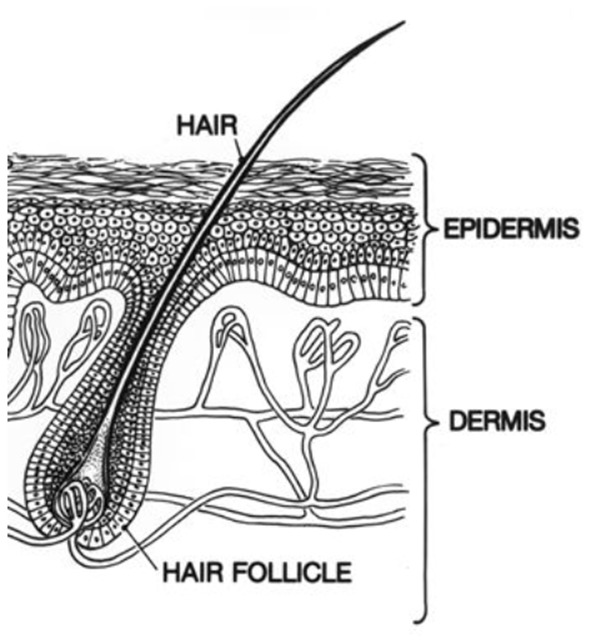
Main parts of skin: epidermal and dermal layers and follicular structures.

Less established is the use of the OCT for the analysis of other biological tissues such as the skin due to the limited resolution and penetration of the light waves within this structure (3). Figure 1 shows the skin structure mainly composed of epidermis and dermis layers. However, during the last decade, this technique has gained great relevance for the study of the skin layers because of the last advances in optical, fiber, and laser technology (4).

Although skin biopsies are currently the gold standard for the diagnosis of a large number of dermatological diseases, this technique is invasive, time-consuming and can cause side effects that make disease monitoring difficult (2, 3). Therefore, the analysis of the main skin structures through non-invasive techniques such as OCT provides a useful non-traumatic alternative to the biopsy.

As well as other quantitative analyses of skin OCTs, the calculation of the epidermal layer thickness, which is important for the diagnosis of several skin disorders, often requires manual segmentation that is very time-consuming and suffers from inter and intra-observer variability. This fact has motivated the development of semi- and fully automated epidermis segmentation methods from OCT images (5–9). Li et al. defined the segmentation of the epidermis in three steps: preprocessing weighted by least squares, detection of the surface of the skin based on graphics, and local integral projection for the detection of the DEJ. The proposed method was evaluated with a dataset composed of five volumes belonging to healthy skin of the arm, obtaining an average overlap ratio of 0.744 (5). Taghavikhalilbad et al. proposed a semi-automated method to detect the location of the DEJ on 115 B-Scan OCT skin images taken from different body parts, obtaining a root-mean-square error between 8 and 14 μ*m*. The proposed method was based on a graphical representation of an attenuation coefficient map through a uniform-cost search method. In addition, for border thinning, a fuzzy-based nonlinear smoothing technique was used (6). Note that the aforementioned works were based on classical image processing techniques. In fact, despite the existence of state-of-the-art methods for segmentation of different skin structures, only few are based on deep learning algorithms (8, 9). Calderon-Delgado et al. proposed a fully convolutional network (FCN) to segment 1756 human skin OCT images into dermis, dermal-epidermal junction, epidermis, glycerol, and glass, obtaining an average accuracy of 88, 91, 90, and 96%, respectively. Note that the main limitation of this work is that complex structures, such as hair follicles, were not segmented (8). Kepp et al. presented a deep learning algorithm based on the U-net architecture which was modified with densely connected convolutions to segment mouse skin OCT volumes. They divided the skin into five classes: (epi-)dermis layer, subcutaneous fat layer, fascia and muscle layer, tattoos and background. They applied their algorithms to 72 B-scans of the mice inguinal region and achieved an average Dice similarity coefficient of 0.86 over all segmented skin structures (9). Note that this work did not differentiate between the epidermis and the dermis region.

After a thorough search of the relevant literature, it was found that no previous studies have been focused on the segmentation of the epidermis layer along with the follicular structures in human skin OCT images. Note that the epidermis detection is of importance in several clinical dermatology applications such as the epidermal thickness determination in healthy versus unhealthy skin (6). In addition, the identification and measurement of pilosebaceous unit and the size and condition of hair follicles can be useful in monitoring several diseases such as lupus erythematosus, folliculitis, alopecia areata, and basal cell carcinomas (BCCs) (10). However, before interpreting pathological processes and identifying skin pathologies using OCT, the healthy skin appearance has to be studied to establish morphological features of normal skin (4, 11). For all of the above, in this paper, a method to detect the epidermis layer with the hair follicles in healthy human skin OCT images is proposed.

## 2. Materials and Methods

### 2.1. Skin Database

The human skin OCT database used in this paper is composed of a total of 270 images from nine healthy humans with ages between 28 and 72 years old (Table 1). The research protocol was approved by the Ethics Committee of the Capital Region of Denmark: no. H-16039077. The skin images were acquired from the cheek of the subjects using a UHR-OCT (ultrahigh-resolution) system developed by the Technical University of Denmark (DTU) (3). Compared with a commercial OCT system, which has a lateral resolution around between 10 and 15 μm and axial resolution of 5-10 μm, the used UHR-OCT system achieves 6 and 2.2 μm for lateral and axial resolution, respectively. Therefore, the UHR-OCT system is able to improve the definition of some structures of the skin images such as follicular structures that are of great importance to detect some skin disorders (3). In terms of image size, the number of image pixels varies from one patient to another (Table 1). In order to be segmented through deep learning techniques, it is required that all images have the same dimension. For this reason, a rescaling of all images to the minimal dimension was performed, in this case, to 526 × 975 pixels.

**Table 1:** Content of the skin OCT database.

**Patients**	**Images**	**Dimensions**
Patient 01	30	601 × 975
Patient 02	30	601 × 975
Patient 03	30	601 × 975
Patient 04	30	526 × 975
Patient 05	30	601 × 975
Patient 06	30	601 × 995
Patient 07	30	601 × 995
Patient 08	30	551 × 995
Patient 09	30	601 × 995

**Figure 2:**
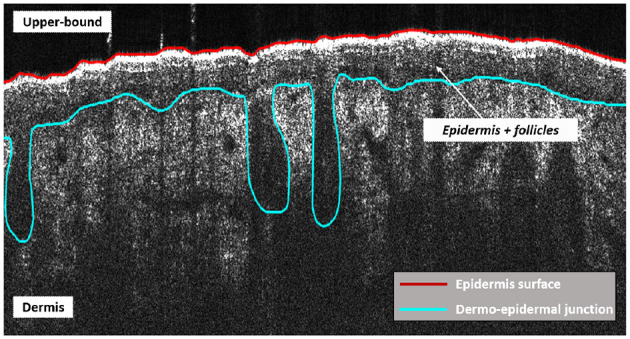
Ground truth of skin layer boundaries.

As was mentioned before, the goal of the method presented in this paper is to segment the epidermis regions as well as the follicular structures, which can be identified in the skin OCT images as variations of the dermo-epidermal junction. These structures were manually segmented by an expert for training and validation purposes (Figure 2). The complete database, including original images and the ground truth, is available as Supplementary Material.

### 2.2. Segmentation Algorithm

In this paper, we present an automatic algorithm able to segment the epidermis regions in OCT volumes from human skin. This algorithm consists of an encoder-decoder fully convolutional network combined with a robust post-processing^1^.

#### 2.2.1. Fully Convolutional Network (FCN)

**Figure 3:**
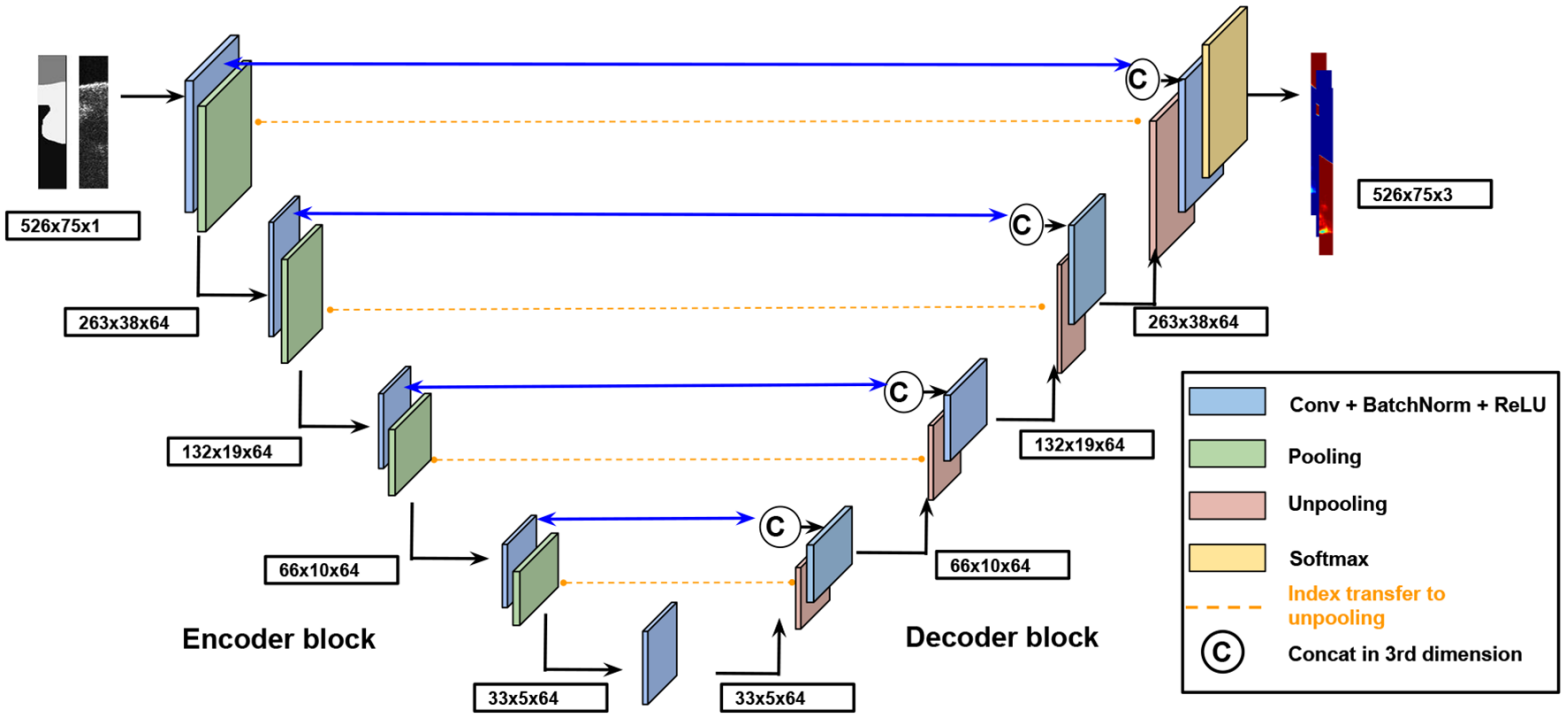
Encoder-decoder architecture proposed to address the segmentation task.

Skin layers segmentation can be interpreted as a classification problem. The aim is to assign each pixel of an OCT image, into its label *l* in the label space *L* = {*l*} = {1, …, λ}. In this work λ = 3, being λ the number of classes (i.e., upper bound, epidermis+follicles, dermis). Note that these layers are shown between the boundaries exposed in Figure 3. The encoder-decoder architecture proposed to address the segmentation task can be observed in Figure 3. The input to the whole algorithm is the original image along with its corresponding ground-truth mask, whereas the outputs are the obtained probability maps, one per class. The architecture components and the training and testing processes will be exposed below.

- ***Encoder-decoder block***

Each encoder block is composed of four layers, in sequence: convolution layer, batch normalization layer, ReLU activation layer and max-pooling layer. The convolutional layer is composed of 64 rectangular kernels of 7 × 3 and zero padding to preserve the spatial dimensions. The batch normalization technique is applied to improve the convergence speed and the performance of the neural network. ReLu activation introduces non-linearities and max-pooling condenses the feature information reducing the spatial dimensions. The pooling indexes of this operation are transferred to the corresponding unpooling layer in the decoder block. In conclusion, the main objective of the encoder part is the extraction of relevant features from the images.

Each decoder block consists of five layers, in sequence: unpooling layer, concatenation layer, convolution layer, batch normalization, and ReLU activation function. The unpooling layer upsamples the feature maps from the previous decoder block to a double resolution by using the achieved pooling indexes to the matched encoder block. After this step, a concatenation of the upsampled feature maps with the corresponding output feature maps is performed. Finally, convolutional layer, batch normalization, and ReLu are applied to the concatenated feature map. The main objective of this part is to collect the characteristics extracted by the encoder to build the output image as accurately as possible and with the same spatial dimension as the input image.

The final decoder block consists of a convolutional layer with 1 × 1 kernel and the softmax activation function. This part is responsible to associate each pixel to one of the three possible classes (upper bound, epidermis+follicles, dermis).

- ***Training process***

**Figure 4:**
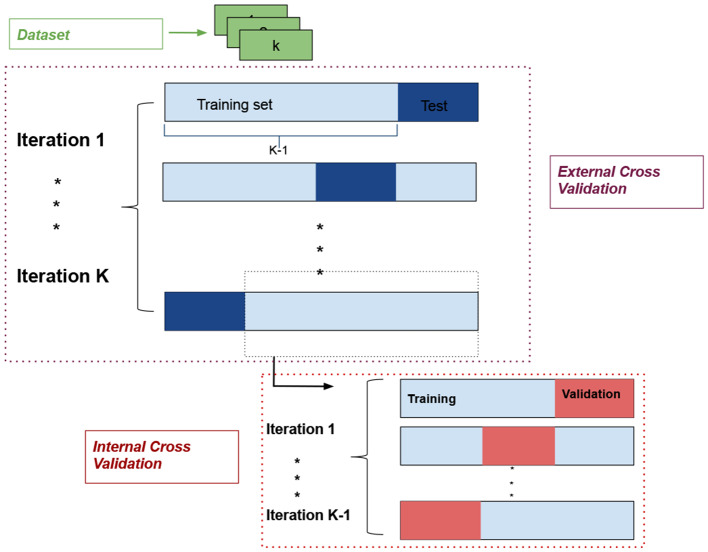
*K*-fold cross-validation technique used to data partitioning.

**Data partitioning**. According to section 2.1, the used dataset is composed of 270 images coming from nine different subjects. To avoid biased results due to a specific partition of the skin OCT images database into training and test subsets and to obtain the segmentation of all images, an external *K*-fold cross-validation technique was carried out. Specifically, *K* = 9 partitions were created, one per patient. Consequently, in the training process, *K*−1 different folds in each external iteration were used, while the remaining partition was utilized to test the model performance. In addition, an internal leave-one-out cross-validation was carried out, using the images from one different training fold in each internal iteration as a validation set (Figure 4).

**Data conditioning**. Because the images used for training have large dimensions, a patch-wise learning methodology was applied in order to avoid memory problems. In this case, patches of size 526 × 75 were used, with a total of 2,730 patches for training and 390 patches for validation. Furthermore, we augmented the sliced data by introducing random geometric transformations such as rotations, horizontal flips, croppings, and translations that permit avoiding the overfitting. In the testing stage, memory requirements were more permissive allowing to predict a test sample into two slices of 526 × 512 with a Titan V GPU.

**Loss function**. The proposed network was trained by optimizing the Soft-Dice loss function. This function evaluates spatial overlap between the algorithm prediction and the ground truth. Since the Soft-Dice loss is a value ranging between 0 and 1, the aim is to maximize this function during the training stage. The used Soft-Dice loss function can be defined as follows:

(1) $$\begin{array}{l} \zeta_{dice}=1-\frac{2\underset{x\in \Omega }{\sum }p_{l}\left(x\right)g_{l}\left(x\right)}{\underset{x\in \Omega }{\sum }p_{l}^{2}\left(x\right)+\underset{x\in \Omega }{\sum }g_{l}^{2}\left(x\right)} \end{array}$$

where *p*_*l*_(*x*) is the predicted multiclass segmentation and *g*_*l*_(*x*) is the ground truth.

**Hyper-parameter configuration**. The proposed network was learned through the stochastic gradient descent (SGD) optimizer. Using this optimizer, each epoch of training was composed of a set of *N* iterations $N=\frac{n^{o}trainingsamples}{BatchSize}$, in which the gradient was calculated only for a batch of training data. In this case, *Batch Size* = 4 and *N* = 683 per epoch. A momentum value of 0.97 was used and the learning rate was initially established to 0.001 and reduced by one order after every 20 epochs. The training stage was composed of 60 epochs and the model that minimizes the validation loss was chosen as the best one.

- ***Testing process***

After training the different models, the images belonging to each patient partition (*K* = 9) were predicted. As was mentioned, an external and internal cross-validation technique was used, obtaining a set of *K* × (*K* − 1) models. Therefore, for the final prediction of each test subset belonging to each patient *P*_*i*_ (*i* = 1, 2, …, *K*), (*K*−1) models were used:

(2) $$\begin{array}{l} P_{i}=\frac{1}{K-1}\overset{K}{\underset{j=1}{\sum }}T_{j}\forall j\neq i \end{array}$$

where *T*_*j*_ is the test subset prediction of each model created with different validation subsets (*j* = 1, 2, …, *K*) with *j* ≠ *i*. The final prediction had a depth equal to the number of classes, so the maximum value in the depth direction was calculated, obtaining for each pixel the class with the highest probability.

#### 2.2.2. Post-processing

**Figure 5:**
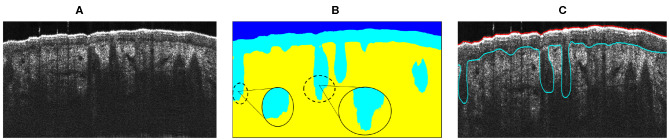
**(A)** Original OCT image. **(B)** Segmentation maps obtained before post-processing. **(C)** Ground truth image.

After testing all skin OCT images of the database (Figure 5A) with the trained models, segmentation maps as those shown in Figure 5B were obtained. As it can be observed in Figure 5C, the proposed network was not able to obtain the characteristic smoothed borders of the follicular structures due to the lack of a pronounced gradient of intensity in its contours. Thus, a post-processing on the obtained segmentation maps was carried out. The implemented post-processing was composed of two main stages that are explained below.

- ***Savitzky-Golay filter***

Hair follicles can be considered invaginations of the epidermis into the dermis. The first step to perform a correct definition of the hair follicles consisted in obtaining the contours of those structures. To achieve this purpose, it was necessary to detect the baseline of the dermo-epidermal signal to discriminate between the part belonging to the epidermis to that belonging to the hair follicles. So that, the Savitzky-Golay (SG) filter was used.

The Savitzky-Golay filter is considered a type of finite response digital filter (FIR) based on the polynomial adjustment of a set of points by means of least squares (12). If a symmetric window of size *N* and centered on point *x*_0_ of a vector **x** of size *N* = 2*M* + 1 is considered, the points contained in this window are symmetric on each side of *x*_0_ as follows:

(3) $$\begin{array}{l} \text{x}={\left[x_{-M},\ldots ,x_{-1},x_{0},x_{1},\ldots ,x_{M}\right]}^{T} \end{array}$$

The *N* samples contained in **x** can be adjusted by a polynomial of order *d* with (0 ≤ *d* ≤ *M*), following the next equation:

(4) $$\begin{array}{l} {\hat{x}}_{m}=c_{0}+c_{1}m+\ldots +c_{d}m^{d}-M\leq m\leq M \end{array}$$

where ${\hat{x}}_{m}$ represents the *m*_*th*_ sample of the smoothing data. The coefficients *c*_*i*_ are real and must be determined in an optimal way, minimizing the least-squares adjustment errors.

(5) $$\begin{array}{l} e=\overset{M}{\underset{m=-M}{\sum }}{\left(x_{m}-{\hat{x}}_{m}\right)}^{2}=\overset{M}{\underset{m=-M}{\sum }}(x_{m}-\left(c_{0}+c_{1}m+\ldots +c_{d}m^{d}\right){)}^{2} \end{array}$$

To obtain the value of these coefficients and therefore the smoothing values, *d*+1 polynomial base vectors represented by **s**_**i**_ (*i* = 0, 1, …, *d*) are defined:

(6) $$\begin{array}{l} {\text{s}}_{\text{i}}\left(m\right)=m^{i}-M\leq m\leq M \end{array}$$

The corresponding **S** matrix (*N* × (*d* + 1)) is established as **S** = [**s**_**o**_, **s**_**1**_, …, **s**_***d***_] and the smoothing values can be organized in a vector as follows:

(7) $$\begin{array}{l} \hat{\text{x}}=\text{B}\text{x}=\overset{d}{\underset{i=0}{\sum }}c_{i}{\text{s}}_{\text{i}} \end{array}$$

where **B** is established as **B** = [**b**_**-*M***_, …, **b**_**0**_, …, **b**_***M***_].

The value $y_{0}=\hat{x_{0}}$ is given in terms of the center of the filter **b**_0_ according to the following equation:

(8) $$\begin{array}{l} y_{0}={\text{b}}_{0}^{T}\text{x}=\overset{M}{\underset{m=-M}{\sum }}b_{0}\left(m\right)x_{m} \end{array}$$

In addition, the N-dimensional vector **x** can be shifted of *n* instants of time as follows:

(9) $$\begin{array}{l} \text{x}={\left[x_{n-M},\ldots ,x_{n-1},x_{n},x_{n+1},\ldots ,x_{n+M}\right]}^{T} \end{array}$$

Therefore, the response of the SG filter with *N* length to smooth a *x*(*n*) signal in a steady-state period can be represented by:

(10) $$\begin{array}{l} y\left(n\right)=\overset{M}{\underset{m=-M}{\sum }}b_{0}\left(-m\right)x\left(n-m\right) \end{array}$$

Note that in input-on and input-off transients periods (at the beginning and the end of the signal), the window goes beyond the limits of the signal and becomes undefined. To handle this problem, in input-on transients periods where *n* = 0, …, *M* − 1:

(11) $$\begin{array}{l} y\left(n\right)=\overset{2M}{\underset{m=0}{\sum }}b_{M-n}\left(m-M\right)x\left(m\right) \end{array}$$

and in input-off transients periods where *n* = *L* − *M*, …, *L* − 1, being *L* the length of the whole signal:

(12) $$\begin{array}{l} y\left(n\right)=\overset{2M}{\underset{m=0}{\sum }}b_{-M-n+\left(L-1\right)}\left(m-M\right)x\left(L-1-2M+m\right) \end{array}$$

The larger the size of the chosen window, the smaller the variance of the error at the filter output. However, if the window is too large, the filter output will be biased compared to the real signal, so the output signal will be very smoothed.

**Figure 6:**
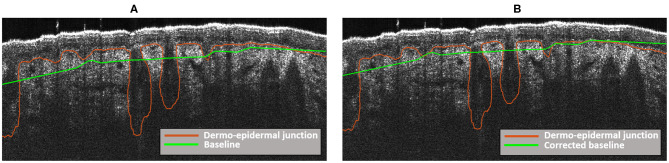
Baseline of the dermo-epidermis junction. **(A)** Baseline obtained after the application of Savitzky and Golay filter. **(B)** Baseline obtained after the correction process.

Since the purpose of applying this method was to obtain the baseline of the dermo-epidermal junction, a polynomial order *d* = 1 and a large window size of 449 pixels were established to obtain the signal as smooth as possible. Note that both the polynomial order and the window size were obtained empirically. The results after the application of this filter can be observed in Figure 6A.

As it is shown in Figure 6A, the obtained baseline of the dermo-epidermal junction (green line) was not completely adjusted at the outset of the three follicular structures content in this image. Therefore, a baseline correction was carried out:

(13) $$\begin{array}{l} bl_{c}\left(x\right)=bl\left(x\right)-dif\left(x\right) \end{array}$$

where *dif*(*x*) = *max*(*s*_*dej*_(*x*))−*max*(*bl*(*x*)) with *s*_*dej*_(*x*) is the signal of dermo-epidermal junction and *bl*(*x*) the baseline obtained after the application of Savitzky-Golay filter. In Figure 6B, the corrected baseline is shown. As it can be observed, the baseline is now more precisely adjusted to obtain the full follicular structures.

The hair follicles are structures located below the baseline of the dermis-epidermis junction obtained. However, after the SG application, not all structures below the obtained baseline are hair follicles. To obtain only the follicular structures, a morphological area opening was performed (13). This operation removes the connected components with an area smaller than a parameter α. In this case, α = 0.3 × max(**A**_**c**_), with **A**_**c**_ being the area of the components below the obtained baseline.

**Figure 7:**
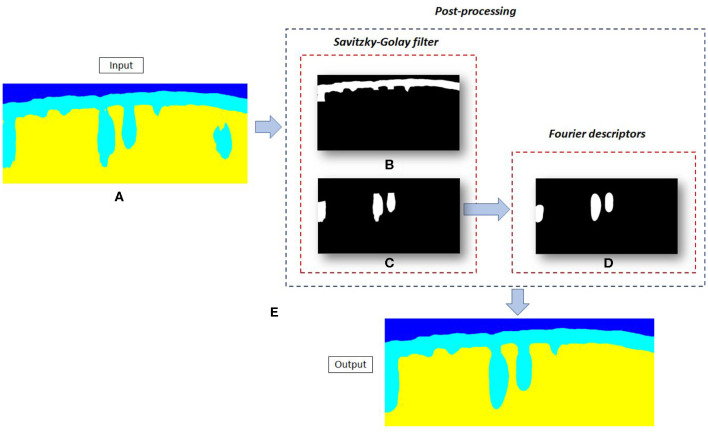
Post-processing. **(A)** Segmentation maps obtained by the fully convolutional network. **(B)** Upper epidermis without follicular structures. **(C)** Follicular structures obtained after the Savitzky-Golay filter. **(D)** Follicular structures softened after the Fourier Domain Filtering application. **(E)** Segmentation maps obtained after post-processing.

After the application of the Savitzky-Golay filter, the upper epidermis region, Figure 7B, and the follicular structures, Figure 7C, were obtained removing any structure that not accomplishes the condition to be a hair follicle as that located on the right side of Figure 7A. These follicular structures are the input to the following post-processing stage.

- ***Fourier Domain Filtering***

After applying the Savitzky-Golay filter to obtain the baseline of the dermo-epidermal junction signal and therefore, detect each follicular structure, a Fourier Domain Filtering is applied to soften their contours. Fourier Domain Filtering is a useful mathematical tool to describe the shape of objects defined by a closed contour line. The first step in the application of the Fourier transform is to obtain the coordinates (*x, y*) of the object contour to be analyzed. The application of the Fourier method requires that the contour was discretized in equispaced coordinates and that the number of contour points *N* was a power of two and belongs to the complex plane:

(14) $$\begin{array}{l} s\left[n\right]=x\left[n\right]+jy\left[n\right] \end{array}$$

where *x*[*n*] and *y*[*n*] are the coordinates of the points of the resampled contour, in this case, the follicular contour.

After obtaining the points of the contour in the complex plane, the Fourier transform is calculated as:

(15) $$\begin{array}{l} S\left[k\right]=\overset{N-1}{\underset{n=0}{\sum }}s\left[n\right]e^{-j\frac{2\pi kn}{N}} \end{array}$$

where *k* = (0, …*N* − 1). After this step, the contour of the object, that in this case is each follicular structure, is transformed into a frequency vector. Since the Fourier transform has inverse, the outline of each object can be reconstructed from its Fourier descriptors. The reconstruction is carried out by applying this equation:

(16) $$\begin{array}{l} s\left[n\right]=\frac{1}{N}\overset{P-1}{\underset{n=0}{\sum }}S\left[k\right]e^{j\frac{2\pi kn}{P}} \end{array}$$

being *P* ≤ *N*. Note that the first descriptors that make up the object, which correspond to the low frequencies, represent the general shape of the contour. However, the latest descriptors, which correspond to high frequencies, represent the finest details. In this case, the roughness of the contour of the hair follicles. Therefore, if the number of descriptors used in the object reconstruction process, *P* decreases, the contour of the object is softened. As the aim of the application of the Fourier Domain Filtering method is the smoothing of the hair follicles and the bandwidth of this signal was approximately 0.06, a frequency cut off (*f*_*c*_) around 0.007 was used. This means that all descriptors with a frequency greater than *f*_*c*_ were removed. Note that the *f*_*c*_ value was heuristically determined. The number of total descriptors used to define the final object can be calculated with the following equation *P* = *f*_*c*_*N*, being *N* equal to the number of total descriptors, about 57 in this case. Therefore, the number of descriptors used to define the hair follicles was *P* = 4. The follicular structures softened after the Fourier Domain Filtering stage are shown in Figure 7D.

Final segmentation maps were obtained by combining the upper epidermis (Figure 7B) with the smoothed follicular structures (Figure 7D). As it can be observed, in Figure 7E, all follicles irregularities have been removed. This allows a better shape definition of the follicular structures, more similar to the manually segmented by experts.

## 3. Results

First, six quantitative measures were computed: Dice's and Jaccard's coefficients, the relative success rate for the number of detected follicles per patient, epidermal thickness, root-mean-square and mean absolute error of the epidermal thickness. Dice's and Jaccard's metrics were calculated for both, the epidermis with hair follicles and the dermis since the layer below the dermo-epidermal junction belongs to the dermis. In addition, these metrics were obtained before and after the post-processing stage to demonstrate the utility of the post-processing in the results of the proposed method (Table 2). As Table 2 demonstrates the improvement of segmentation by including post processing (bold values), from this point, the remaining metrics will be computed on the segmentation results after post-processing.

**Table 2:** Dice's and Jaccard's metrics (mean and standard deviation) comparing the results of the proposed method before and after post-processing (PP) with the ground truth.

**Layers**	**Dice**		**Jaccard**	
	**Before PP**	**After PP**	**Before PP**	**After PP**
Epidermis + follicles	0.81 ± 0.06	**0.83**±**0.06**	0.69 ± 0.09	**0.71**±**0.09**
Dermis	0.95 ± 0.01	**0.96**±**0.01**	0.92 ± 0.02	**0.93**±**0.02**

The relative success rate (RSR) for the number of the detected follicles was calculated with the following equation:

(17) $$\begin{array}{l} RSR=1-\frac{\left|F_{GT}-F_{P}\right|}{F_{GT}} \end{array}$$

where *F*_*GT*_ and *F*_*P*_ are the number of follicles detected by the ground truth and by the proposed method, respectively (Table 3). To obtain the follicular structures, the Savizky-Golay filter was used. After the application of the SG filter and the morphological area opening explained in section 2.2.2, the follicular structures of the ground truth and the proposed method were obtained per patient. After that, the connected components were automatically counted.

**Table 3:** Number of detected follicles comparing the results of the proposed method with the ground truth (GT).

**Patients**	***F*_*GT*_**	***F*_*P*_**	**RSR**
Patient 01	5	5	1.00
Patient 02	3	4	0.67
Patient 03	5	6	0.80
Patient 04	4	5	0.75
Patient 05	5	5	1.00
Patient 06	7	7	1.00
Patient 07	3	3	1.00
Patient 08	4	6	0.50
Patient 09	4	4	1.00
Mean total			0.86

For the epidermal thickness (ET), the average distance between the dermo-epidermal junction (DEJ) and the epidermis surface is considered. The dermo-epidermis junction without hair follicles was calculated for this measure because, according to experts' knowledge, it is better to consider the thin region of the epidermis for the diagnosis of disorders associated with loss of epidermal thickness (11, 14). The hair follicles were manually removed from the GT by experts. The averaged values as well as standard deviation of ET for each healthy subject are reported and compared with the values of the ground truth (Table 4). The three lowest values of RSME and MAE are shown in bold.

**Table 4:** Epidermis thickness (ET) comparing the results of the proposed method with the ground truth (GT).

**Patients**	**GT-based ET (μm)**	**Proposed-method-based** **ET (μm)**	**RMSE** **(μm)**	**MAE** **(μm)**
Patient 01	56.06 ± 2.05	65.02 ± 0.76	9.13	8.95
Patient 02	49.20 ± 1.50	58.19 ± 1.77	9.28	8.98
Patient 03	78.90 ± 3.83	62.50 ± 2.75	**8.90**	**8.68**
Patient 04	82.47 ± 2.63	87.00 ± 1.90	**5.75**	**4.62**
Patient 05	71.30 ± 3.83	83.65 ± 3.02	13.29	12.9
Patient 06	71.43 ± 2.40	77.90 ± 1.99	**7.30**	**6.61**
Patient 07	92.12 ± 3.37	106.00 ± 1.20	14.40	13.87
Patient 08	76.12 ± 1.67	85.42 ± 0.76	9.70	9.29
Patient 09	85.16± 2.94	98.69 ± 1.18	14.10	13.52
Mean total	73.60 ± 2.69	80.50 ± 1.70	10.20	9.76

The root-mean-square error (RMSE) and the mean absolute error (MAE) were calculated with the following equations:

(18) $$\begin{array}{l} RMSE=\sqrt{\frac{\sum_{k=1}^{N}{\left(G\left[k\right]-P\left[k\right]\right)}^{2}}{N}} \end{array}$$

(19) $$\begin{array}{l} MAE=\frac{1}{N}\overset{N}{\underset{k=1}{\sum }}|G\left[k\right]-P\left[k\right]| \end{array}$$

where in both metrics *G* and *P* are the ET obtained by experts and the prediction of the proposed method, respectively, and *N* is the number of OCT images.

**Figure 8:**
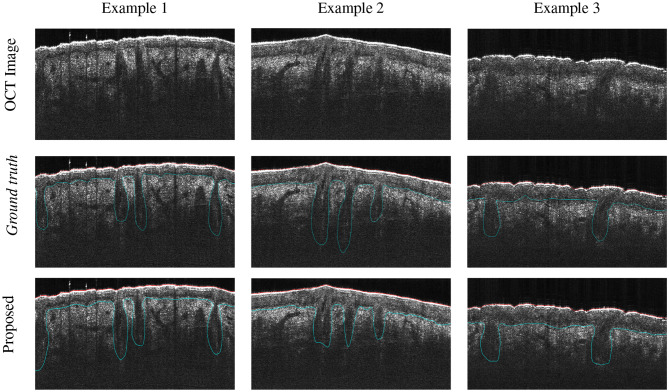
Segmentation results (including post-processing) on three representative examples of human skin OCT images. Color code: skin surface (red), dermo-epidermal junction along with hair follicles (blue).

Secondly, for qualitative evaluation, Figure 8 shows three examples of representative segmentation results obtained by the proposed method.

## 4. Discussion

**Figure 9:**
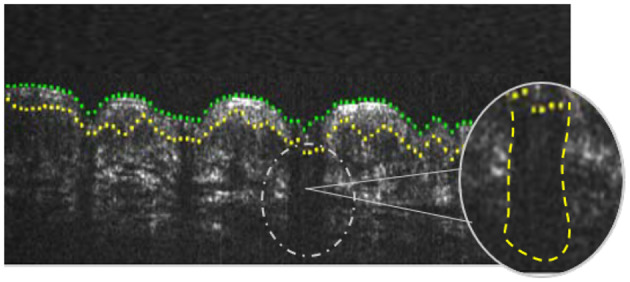
Segmentation of follicular structures in another state-of-the-art work. Note that the method misdetect the hair follicles. The area where follicles should be detected is marked and zoomed in with the correct segmentation. Image directly extracted from Li et al. (5).

In this work, we presented an automatic algorithm for the segmentation of the epidermis along with the follicular structures in healthy patients. As no previous studies have been based on the detection of follicular structures, the results of Dice's and Jaccard's coefficients obtained in this study cannot be extrapolated and reliably compared with those existing in the state of the art. However, there are some studies focused only on the segmentation of the epidermal layer (5). It satisfactory segments the epidermis but fails in the detection of the hair follicles (Figure 9). In particular, the method presented by Li et al. (5) obtains a Dice's coefficient of 0.74 in the segmentation of the epidermis layer in contrast to the 0.83 obtained by the proposed method that segments the epidermis along with follicular structures, which adds considerable difficulty.

Regarding the amount of follicles detected per patient, the proposed method achieves a mean relative success rate of 0.86, which demonstrates that the proposed algorithm successfully detects the follicles structures from patients with different ages.

With respect to the measurement of epidermal thickness, only the epidermis region was considered because hair follicles were not considered relevant enough in this case. Compared to the RSME error provided by another semi-automatic method of the state of the art, the proposed method achieves an error of 10.2, while (6) obtains an error between 8 and 14 μm. Note that this is an indirect comparison since the epidermal thickness was calculated on two different databases focused on different body parts and acquired with different OCT devices. However, in addition to be a fully automatic method, the error presented by the developed algorithm is among the lowest errors obtained by the semi-automatic method which were body-part dependent (6). Note that no publicly available skin OCT segmentation method that can be tested on our images to perform a direct comparison was found because most methods designed for this goal are not open access.

As commented in section 1, the detection of follicular structures is of importance in some skin disorders. For example, basal cell carcinoma, the most prevalent cancer in Caucasians human, is related to hair follicles. In fact, several studies confirm that BCCs arise from basal cells specifically of the hair follicles (15, 16). Therefore, in this case, the obtaining of the hair follicles in the OCT images would be useful to study the changes in follicular volume and size produced by cancerous effects. The follicular structures are also the main target in a subtype of cutaneous lupus erythematosus (LE) called chronic discoid LE (CDLE), which results in an irreversible hair loss due to the recruitment of the cells to the bulge region of the hair follicles coupled with the collapse of immune privilege ultimately (17).

Acne is another disorder related to alterations in hair follicles that is easily detected in OCT images (18). Several studies demonstrate that the dynamics of the acne skin are characterized by the increase in the number of dysmorphic pilosebaceous units and the hyperkeratinization of the acroinfundibulum of the pilosebaceous duct before the occurrence of inflammatory events around the follicle. Furthermore, superficial inflammation of the hair follicles in the epidermis is also produced when folliculitis occurs (19). The OCT images show an ill-defined border of the follicular structures in this type of disorder (20).

Due to the fact that the algorithm presented in this work allows to detect hair follicles and distinguish them from the epidermis in an automatic way, it could be of interest to the analysis of diseases such as those mentioned above. The detection of hair follicles carried out by the proposed algorithm could be used to detect a significant decrease in the number of follicles of a patient during a period of time. In addition, the obtaining each follicular structure would also facilitate a further automatic analysis and comparison of its shape and area with a reference pattern which is useful in diseases that cause a significant change in the follicular structures.

## 5. Conclusion

OCT analysis in human skin is of great relevance because, compared to other invasive techniques such as biopsies, the OCT allows to capture the most important skin structures without perforation. This fact is especially important for the follow-up of dermatological treatments or the detection of skin tumors. However, before analyzing pathological images, it is necessary to study images from healthy patients to establish reference markers to be compared. In that context, this paper proposes an approach to segment the epidermal layer along with follicular structures in healthy human OCT images. The proposed algorithm is composed of two main stages. The first stage is a CNN based on U-Net architecture. The second stage is a robust post-processing based on a Savitzky-Golay filter and Fourier Domain Filtering. The Savitzky-Golay filter is used to obtain the baseline of DEJ junction and detect the hair follicles. The Fourier Domain Filtering is used over the hair follicles to smooth their contour. To the best of the authors' knowledge, this approach is the only epidermis detection algorithm that segments the follicular structures, which are of importance in the progress of several skin disorders. So, the introduction of the proposed method into clinical practice would accelerate the process of segmentation some important skin structures, allowing the early diagnosis of several skin disorders in a near future. With the aim of facilitating further comparisons and avoid having to use different databases to evaluate the goodness of the segmentation method, the dataset used in this work along with their ground truth (expert-reviewed segmentation) were made publicly as Supplementary Material.

From a technical perspective, the future lines of work will focus on adapting the network architecture, if it is necessary, to segment pathological images of different disorders. In this way, a distinction between healthy and pathological images would be carried out.

## Data Availability Statement

All datasets generated for this study are included in the article/Supplementary Material.

## Ethics Statement

The studies involving human participants were reviewed and approved by the Ethics Committee of the Capital Region of Denmark: no. H-16039077. The patients/participants provided their written informed consent to participate in this study.

## Author Contributions

RA is the corresponding author. She is the specialist researcher in image processing and the main author of this work. AC is the specialist researcher in deep learning. He guided RA to investigate deep learning techniques and develop the proposed algorithm. SM supervised and reviewed the manuscript carefully and she gave helpful tips to RA for the manuscript writing. MM is the dermatology specialist and brought her expertise about the skin and provided interesting comments. MJ is a specialist in OCT image refinement and has been in charge of the shadow compensation algorithm and contributed to the numerical dispersion compensation. Both methods were applied for refinement of the UHR OCT images in terms of improved contrast and improved resolution. NI is the specialist researcher in OCT and he constructed the UHR-OCT system and provided the UHR-OCT images. Additionally, he helped in defining the results provided for the proposed algorithm. OB is the specialist researcher in charge of UHR-OCT system development. He provided the used database. VN is the lead researcher, she defined the goal of this work, and participated in the revision of the final manuscript providing interesting comments and ideas.

## Conflict of Interest

The authors declare that the research was conducted in the absence of any commercial or financial relationships that could be construed as a potential conflict of interest.

## Footnotes

^1^Request for materials should be addressed to RA (email: madeam2@upv.es).
